# Vimentin-Rab7a Pathway Mediates the Migration of MSCs and Lead to Therapeutic Effects on ARDS

**DOI:** 10.1155/2021/9992381

**Published:** 2021-07-29

**Authors:** Kai Wang, Boxiang Du, Yan Zhang, Congyou Wu, Xiuli Wang, Xu Zhang, Liwei Wang

**Affiliations:** ^1^School of Medicine, Tongji University, Shanghai 200092, China; ^2^Department of Anesthesiology, Xuzhou Central Hospital, Jiangsu 221009, China; ^3^Department of Anesthesiology, The Second Affiliated Hospital of Nantong University, Jiangsu 226000, China

## Abstract

Acute respiratory distress syndrome (ARDS) is difficult to treat and has a high mortality rate. Mesenchymal stem cells (MSCs) have an important therapeutic effect in ARDS. While the mechanism of MSC migration to the lungs remains unclear, the role of MSCs is of great clinical significance. To this end, we constructed vimentin knockout mice, extracted bone MSCs from the mice, and used them for the treatment of LPS-induced ARDS. H&E staining and Masson staining of mouse lung tissue allowed us to assess the degree of damage and fibrosis of mouse lung tissue. By measuring serum TNF-*α*, TGF-*β*, and INF-*γ*, we were able to monitor the release of inflammatory factors. Finally, through immunoprecipitation and gene knockout experiments, we identified upstream molecules that regulate vimentin and elucidated the mechanism that mediates MSC migration. As a result, we found that MSCs from wild-type mice can significantly alleviate ARDS and reduce lung inflammation, while vimentin gene knockout reduced the therapeutic effect of MSCs in ARDS. Cytological experiments showed that vimentin gene knockout can significantly inhibit the migration of MSCs and showed that it changes the proliferation and differentiation status of MSCs. Further experiments found that vimentin's regulation of MSC migration is mainly mediated by Rab7a. Rab7a knockout blocked the migration of MSCs and weakened the therapeutic effect of MSCs in ARDS. In conclusion, we have shown that the Vimentin-Rab7a pathway mediates migration of MSCs and leads to therapeutic effects in ARDS.

## 1. Introduction

Acute respiratory distress syndrome (ARDS) refers to diffuse lung injury caused by severe infection, trauma, shock, or surgery. The clinical manifestations are acute episodes of dyspnea and refractory hypoxia. The current incidence of hyperemia and acute respiratory failure in adults is about 17.6-64 per 100,000 annually [1]. The disease is characterized by rapid onset and progress and is relatively difficult to treat. Treatment consists mainly of protective lung ventilation strategies and early fluid resuscitation. Despite the continuous improvement in treatment methods, the mortality rate remains as high as 40%-60% [2]. The lack of specific etiological treatment measures is an important factor in the high mortality rate of ARDS. The pathogenesis of ARDS is complicated. Its main pathophysiological changes are damage to alveolar capillary endothelial cells and alveolar epithelial cells, which leads to increased alveolar membrane permeability, causing a large amount of protein-rich fluid to leak out of the lung interstitium and alveoli. Effectively repairing these damaged cells is the key to the early treatment of ARDS.

Mesenchymal stem cells (MSCs) are pluripotent stem cells isolated from various tissues, such as bone marrow, fat, placenta, and umbilical cord blood. They can be induced to differentiate, in vitro, into bone cells, chondrocytes, and chondrocytes. A variety of tissue cells such as muscle cells, fat cells, and fibroblasts. When tissues, such as muscle, fat, and fibroblasts, are damaged, MSCs quickly migrate to the damaged site, induce regeneration into normal cells at the site, and participate in tissue repair. MSCs can also secrete a variety of cytokines to exert anti-inflammatory and repair effects. In recent years, a large number of studies have shown that MSCs can effectively treat a variety of diseases, including myocardial injury and diabetes [1, 2]. In ARDS, MSCs exert a significant therapeutic effect, both in animals and humans, showing its unique and excellent anti-inflammatory and antidamage properties [3, 4]. However, the mechanism of MSC treatment in ARDS is not clear, particularly the molecular mechanism of MSC migration to the lung injury site.

Vimentin is a cytoskeletal protein that belongs to the type III intermediate filament family. The relative molecular weight of vimentin protein is 57 kD, and the phosphorylation site in the domain is the key to powering vimentin and conducting molecular signals [5]. Vimentin plays an important role in cell migration, contraction, proliferation, protein synthesis, gene expression, cell apoptosis, and mechanical force transmission, among which participation in cell migration is an important prerequisite for tissue damage repair and inflammation control [6, 7]. Some studies have confirmed that vimentin is regulated mainly by Rab7a, a small molecule GTP binding protein. Under the combined action of the Vimentin-Rab7a pathway, cells can migrate, which mediates a variety of biological effects [8, 9]. Therefore, we speculated that Vimentin-Rab7a plays an important role in mediating the migration of MSCs to the damaged lung tissue and the treatment of ARDS, and this aspect of work has not yet been reported.

In this study, we found for the first time that vimentin mediated the migration and colonization of MSCs in damaged lung tissues, thereby playing a role in the treatment of ARDS, and elucidated the mechanism showing that vimentin is regulated by Rab7a to effect the migration of MSCs.

## 2. Materials and Methods

### 2.1. Animals, Drugs, Reagents, and Instruments

C57BL/6 (male, 6-8 weeks old) mice weighing 22-25 g were used in this study. Animal breeding conditions meet the requirements of the Experimental Animal Welfare Ethics Committee. The mice had free access to food and water. Mice were housed in an adaptive feeding environment with alternating light and dark for 12 hours, starting after 1 week experiment, at 23.0-25.0°C and 55% ~ 65% relative humidity. Vimentin, Rab7a, and GAPDH (Cat. Nos.: ab92547, ab255423, and ab8245) were all purchased from Abcam (Cambridge, MA, USA).

### 2.2. Establishment of LPS-Induced ARDS Model

The animal experiment protocol of this study complies with the requirements of the US Guidelines for the Management and Use of Laboratory Animals. The establishment of the mouse LPS-ARDS model refers to the method previously reported in the literature [10]. After the mice were anesthetized by intraperitoneal injection of 50 mg/mL pentobarbital, 50 *μ*L of LPS with a concentration of 2 mg/mL was injected into the airway under the direct vision of a microinjector to establish an LPS-induced ARDS model, and the objects were inhaled with pure oxygen through a mask until fully awakened.

### 2.3. Experiment Grouping and Processing

After 1 week of adaptive feeding, the C57BL/6 mice were randomly divided into 3 groups, 10 in the normal control group (given the same dose of normal saline intravenously), 10 in the LPS-induced ARDS group, and 10 in the ARDS+MSC treatment group. The tail vein of each experimental animal was injected with 1 × 10^6^ MSCs. At 1, 2, 4, and 16 weeks after LPS-induced ARDS, mouse lung tissues were collected for H&E staining, Masson staining, and *α*-SMA immunohistochemical staining. Concentrations of TNF-*α*, TGF-*β*, and INF-*γ* were measured in blood collected from the endocanthal vein.

### 2.4. H&E Staining

Mouse lung tissue was taken from the mice, fixed dehydrated, and embedded in paraffin. The samples were sliced into 5 *μ*m sections. 4 slices from each group were dewaxed with xylene and rehydrated using an ethanol gradient. The nuclei were stained with hematoxylin, and eosin was used to stain the cytoplasm. Following routine dehydration and washing, the samples were sealed with a neutral gum sheet. The specimens were numbered, and the pathological changes of lung tissue structure were observed through an optical microscope. Three specimens were selected from each group, and eight fields were randomly selected under an optical microscope at 400x magnification. The degree of lung tissue damage was quantitatively analysed according to the Ashcroft score [11].

### 2.5. Masson Staining

Masson trichrome staining was performed using the Trichrome Stain (Masson) Kit (Sigma-Aldrich, St. Louis, MO, USA). Ashcroft scores were blindly assigned based on a modified system of grades using slides stained with Masson trichrome [12].

### 2.6. MSC Isolation and Culture

After C57BL/6 mice were sacrificed and disinfected, they were placed on a clean bench to separate mouse femurs. Bone marrow was washed thoroughly using the medium and placed in a petri dish. According to the experimental design, the washed cells were cultured in an incubator at 37°C incubator with 5% CO_2_. Cell growth was observed, and cells were photographed daily. The cells were passaged when they reached 70-80% confluency.

### 2.7. Detection of MSC Surface Markers by Flow Cytometry

MSCs were dissociated with trypsin and centrifuged to concentrate. The cells were resuspended in PBS to a concentration of approximately 1 × 10^6^ cells/mL. 100 *μ*L of cells were transferred to another EP tube, and the appropriate amount of antibody was added (2 *μ*L each for CD105, CD90, CD31, and CD45). The cells were incubated with antibody for 30 min in the dark at RT. 500 *μ*L of Stain Buffer was added to each tube; the sample cell count was acquired by flow cytometry.

### 2.8. GFP Labeled MSCs

2 × 10^5^/mL MSCs were plated in a petri dish to prepare for transfection. When the cells reached 70-80% confluency, they were transfected using polybrene and 6 *μ*g/mL GFP viral vector. After 18-20 hours, the culture medium was changed. After 24 hours, the cells were photographed under a fluorescence microscope (excitation at 488 nm), and the number of fluorescent cells and fluorescence intensity was measured.

### 2.9. Enzyme-Linked Immunosorbent Assay (ELISA Experiment)

The double antibody sandwich ABC-ELISA method was used to detect the corresponding cytokines in mouse serum. Coated on the ELISA plate, the standard and corresponding target molecules in the samples which were to be tested were combined with the corresponding monoclonal antibody in the ELISA plate, which was coated with anti-mouse TNF-*α*, TGF-*β*, INF-*γ*, and other cytokine monoclonal antibodies. The sample was subsequently incubated with the corresponding secondary antibody. The primary antibody and the secondary antibody combined to form an immune complex and adhere to the plate. HRP-labeled streptavidin bounds to biotin. The substrate working solution was added to produce a blue color, and the reaction was stopped with the addition of sulfuric acid. The absorbance (450 nm) was then measured in each well. The OD values were proportional to the concentration of the molecules of interest, and the corresponding cytokine concentration in the sample was calculated against a standard curve.

### 2.10. Western Blotting

Cells or tissues were lysed with RIPA (Beyotime Biotechnology, China) and subjected to protein concentration determination using BCA assay. The obtained protein samples were loaded at 40 mg/well for SDS-PAGE. Proteins were electrotransferred onto a PVDF membrane at a constant 70 V. The PVDF membrane was placed into blocking solution containing 5% skim milk for 2 hours at room temperature and incubated with primary antibody solution overnight at 4°C. The membrane was washed and incubated with secondary antibody for 2 hours. Protein bands were visualized via ECL.

### 2.11. Statistics

All experiments were repeated three times to ensure the reliability of the results. All data were presented as mean ± SD. SPSS version 19.0 (IBM Corp, Armonk, NY, USA) was used for statistical analysis. The independent sample *t*-test was used for comparison between the two groups; *P* < 0.05 indicated significant differences.

## 3. Results

### 3.1. Construction of LPS-Induced ARDS Model in Mice

**Figure 1:**
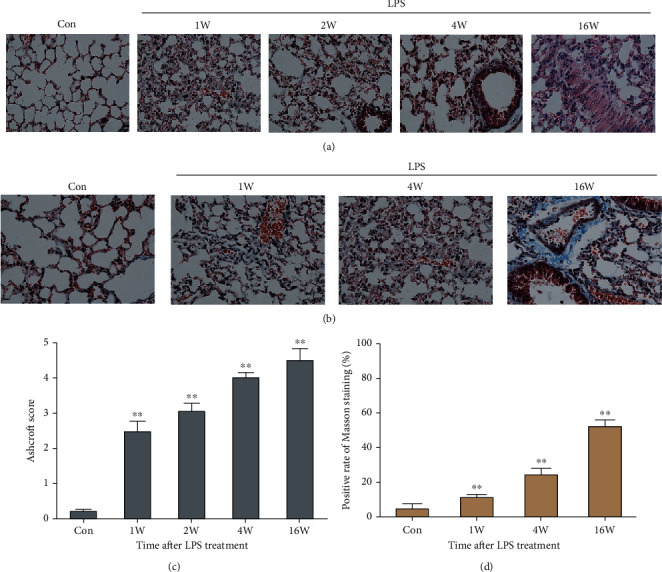
Construction of LPS-induced ARDS model in mice. 50 *μ*L of LPS at a concentration of 2 mg/mL was injected into the airway of mice. Left lung tissues of mice were taken at 1 W, 2 W, 4 W, and 16 W for H&E staining (a) and quantified using the Ashcroft score (c). Masson staining was used to assess the degree of fibrosis in the left lung tissue at 1 W, 4 W, and 16 W after LPS-induced ARDS (b) and quantified by calculating the blue stained area (d). Statistical results are represented by mean ± SD. ^∗∗^*P* < 0.01, *n* = 10.

We constructed an LPS-induced acute lung injury model in mice according to the method of Rittirsch et al. [10]. After induction of LPS (2 mg/mL) tracheal instillation, H&E and Masson staining were performed at 1 W, 2 W, 4 W, and 16 W to assess pneumonia and pulmonary fibrosis in mice. The results showed that LPS can induce lung tissue damage and small amounts of inflammation. The manifestations included lung tissue congestion, edema, alveolar exudation, increased red blood cells, and increased inflammatory cell infiltration. By the 4th and 16th weeks, the alveolar separation was obvious thickening, which indicated an increase in the degree of nonfibrosis (Figures 1(a) and 1(c)). Additionally, Masson staining (blue indicated increased collagen) showed that the lung septum of mice gradually thickened after LPS induction, and the blue area also gradually increased. The blue area increased significantly, especially at the 16th week, which indicated that the degree of fibrosis was increased (Figures 1(b) and 1(d)). The above results indicated that the LPS-induced acute lung injury model in mice was successfully constructed.

### 3.2. MSCs Effectively Treated ARDS in Mice

**Figure 2:**
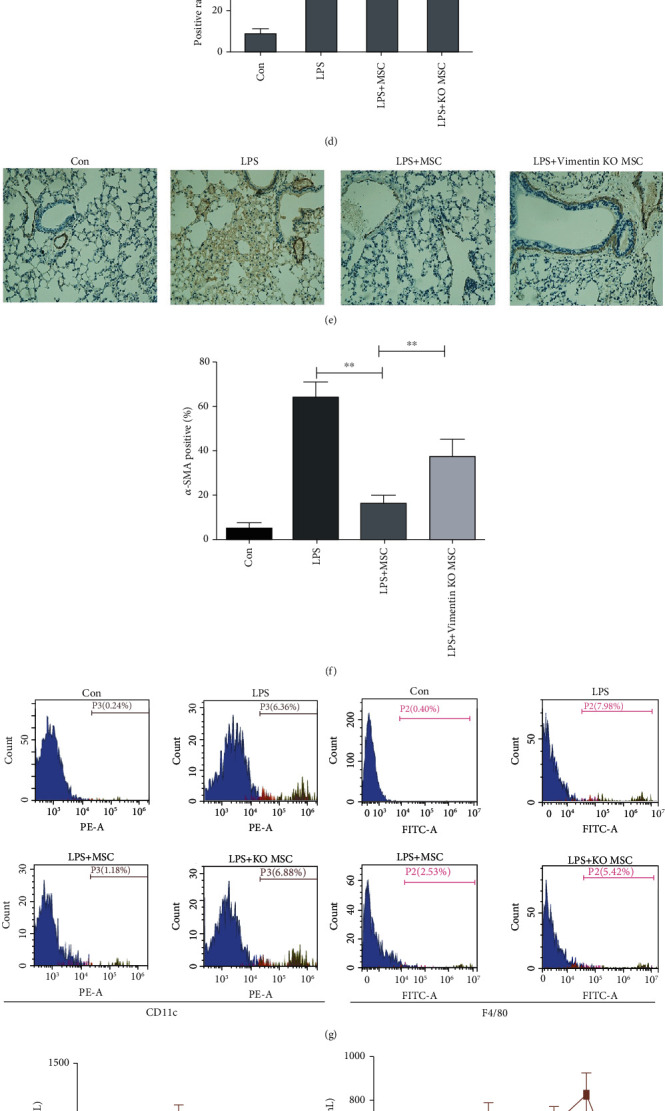
Vimentin knockout alleviated the therapeutic effect of MSCs on ARDS. Two hours after inducing ARDS in mice, MSCs from wild-type mice and vimentin knockout mice were injected, and the mice were housed for 1 W, 4 W, and 16 W. Lung tissue was stained by H&E (a) and quantified by calculating alveolar thickness (b). Masson staining (c, d) and *α*-SMA immunohistochemical staining (e, f) were performed on the 16 W lung tissue to assess the degree of pulmonary fibrosis. On the second day after LPS induction of ARDS, the alveolar lavage fluid of each group of mice was collected, labelled with CD11c and F4/80, and then counted via flow cytometry (g) to clarify the differentiation of MSCs. In order to observe the effect of MSCs on the release of ARDS inflammatory factors, the peripheral serum of mice was collected at 1 D, 3 D, 5 D, 1 W, 2 W, 4 W, 8 W, and 16 W after LPS-induced ARDS, and TNF-*α* (h), TGF-*β* (i), and INF-*γ* (j) contents were tested. Two hours after the ARDS model was constructed, MSCs from GFP-labeled wild-type mice and vimentin knockout mice were injected through the tail vein of the mouse. At 24 h, 48 h, 72 h, and 96 h, the left lung tissue of the mouse was obtained and observed under a British crown microscope. GFP-labelled cells (k). Statistical results are represented by mean ± SD. ^∗^*P* < 0.05, ^∗∗^*P* < 0.01, *n* = 5.

Based on the establishment of the LPS-induced acute lung injury model in mice, we further studied the treatment of ARDS with MSCs. Mice were infused with MSCs for 1 hour, and lung tissues were taken at 1, 4, and 16 weeks to observe the damage degree. The results show that MSCs can effectively reduce LPS-induced lung injury, lung congestion, and edema and relieve the exudation of inflammatory cells (Figures 2(a) and 2(b)). Masson staining and *α*-SMA staining also showed that the treatment of MSCs can reduce tissue damage. The positive area of Masson (Figures 2(c) and 2(d)) and *α*-SMA (Figures 2(e) and 2(f)) indicated that MSCs effectively alleviated pulmonary fibrosis.

### 3.3. Vimentin Knockout Inhibited the Proliferation of MSCs and Changed the Differentiation State of MSCs

**Figure 3:**
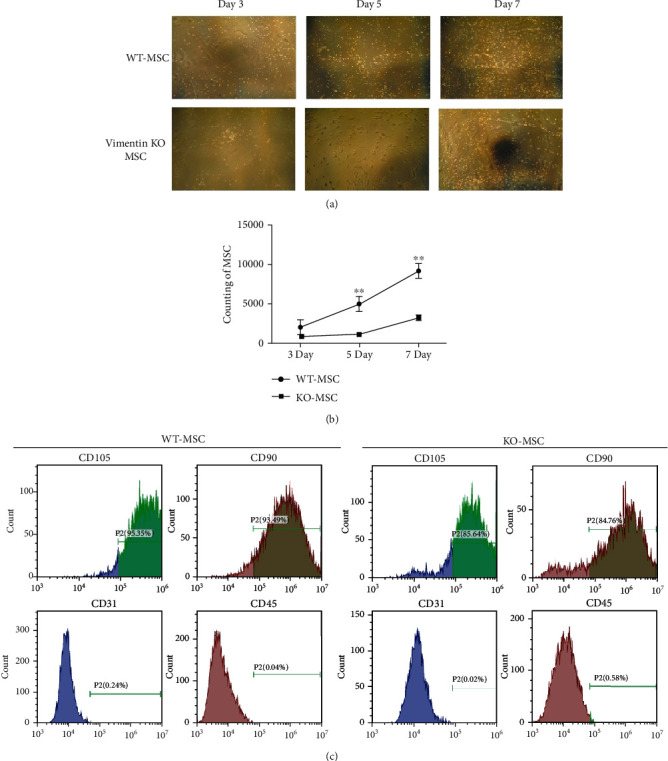
The effect of vimentin knockout on the characteristics of mouse MSCs. MSCs were isolated and cultured in vitro in wild-type mice and vimentin gene knockout mice. On the 3rd, 5th, and 7th day after culture, the morphological changes were examined via microscopy (a) and counted (b) at the same time. The MSCs cultured to the 7th day were labelled with CD105, CD90, CD31, and CD45, and the relevant expression levels were detected by flow cytometry. Statistical results are represented as mean ± SD. ^∗^*P* < 0.05, ^∗∗^*P* < 0.01, *n* = 3.

In order to confirm the key role of vimentin in mediating MSC treatment of ARDS, we constructed a vimentin knockout mouse model. We tested the difference in proliferation and differentiation between MSCs and wild-type mouse MSCs of vimentin knockout mice. The results showed that under the conditions of in vitro culture, the growth of vimentin knockout mouse MSCs was significantly weaker than normal MSCs from day 5, indicating that vimentin knockout had a certain effect on the proliferation of MSCs (Figures 3(a) and 3(b)). MSCs were labelled with CD105, CD90, CD31, and CD45, and it was found that MSCs of wild-type and vimentin gene knockout mice were positive for CD105 and CD90, while CD31 and CD45 were negative, indicating that the purity of the obtained MSCs was relatively high. However, the positive rates of CD105 and CD90 in MSCs of vimentin gene knockout mice were significantly lower than those of wild-type MSCs, suggesting that vimentin gene knockout had a certain effect on the differentiation of mouse MSCs.

### 3.4. Vimentin Knockout Attenuated the Therapeutic Effect of MSCs on ARDS in Mice

We compared the therapeutic effects of wild-type mouse MSCs and vimentin knock-out mouse MSCs on LPS-induced ARDS. The results showed that, compared with wild-type MSCs, the MSCs of vimentin knockout mice had a significantly weaker therapeutic effect, showing increased edema and inflammatory cell exudation (Figures 2(a) and 2(b)) and significantly increased the degree of fibrosis (Figures 2(c)–2(f)). Mouse alveolar lavage fluid was collected on day 2 after LPS induction, and flow cytometry was used to detect the content of F4/80 positive macrophages. On day 2 after LPS induction, alveolar CD11b and F4/80 were found to be increased, while the MSC treatment group decreased, indicating that MSCs can reduce lung inflammatory cell infiltration caused by LPS, but the knockout of vimentin offsets the protective effect of MSCs (Figure 2(g)). TNF-*α* and TGF-*β* are important indicators of LPS-induced pneumonia, and their increase can reflect the state of pneumonia, especially as TGF-*β* is considered to be important for fibrosis. Experimental results showed that TNF-*α* increased with time after LPS induction, reached a peak at 1 W, and then dropped. Meanwhile, TGF-*β* began to increase after 1 W, and the increase was more obvious as time progressed. As a protective factor for pneumonia, especially fibrosis, INF-*γ* also increased in the late stage of LPS induction. The above results are consistent with the typical manifestations of LPS-induced pneumonia. On this basis, the use of different MSC treatments found that wild-type mouse MSCs can block the increase of TNF-*α* and TGF-*β* and increase the content of INF-*γ* in the later stage, but the effect of vimentin knockout mouse MSCs is reduced (Figures 2(h)–2(j)). After two mouse MSCs were labelled with GFP, they were injected into the tail vein of LPS-induced ARDS mice, and lung tissues were taken at different times to observe the fluorescent (green) signal. Wild-type mouse MSCs were detected in the lungs at 24 h, peaked at 48 h, and decreased after 96 h. Meanwhile, the MSCs of vimentin knockout mice were less colonized, indicating that vimentin plays an important role in MSC homing (Figure 2(k)).

### 3.5. Rab7a Affects the Migration of MSCs by Acting on Vimentin

**Figure 4:**
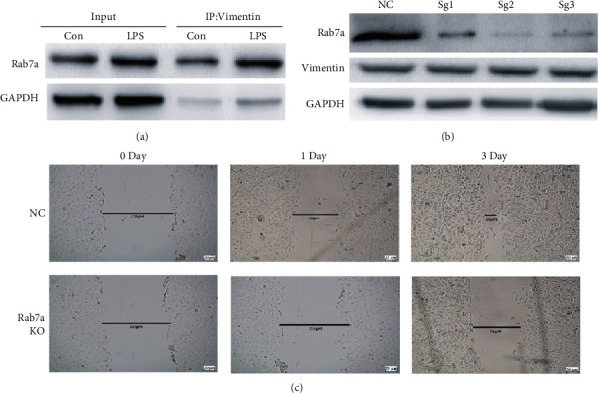
Rab7a knockout inhibited the migration of MSCs. In order to detect the binding of Rab7a and vimentin, the state of Rab7a contained in vimentin was detected by immunoprecipitation method (a). Lentiviral transfection was used to knock out Rab7a and confirm knockout (b). The scratch test was used to determine the impact of Rab7a knockout on scratch healing, a measure of cell migration capability (c).

As the upstream regulator of vimentin [8, 9], Rab7a has been proven to play an important role in cell migration. We explored whether the Vimentin-Rab7a pathway affects the migration of MSCs. First, we verified the combination of Rab7a and vimentin using immunoprecipitation and found that Rab7a was detected in the precipitated vimentin, and its expression was upregulated under the induction of LPS. It shows that vimentin bound to Rab7a, and LPS upregulated the expression of Rab7a (Figure 4(a)). Furthermore, we constructed Rab7a knockout MSCs (Figure 4(b)) and used the scratch test to determine the effect of Rab7a on the migration ability of MSCs. The results showed that compared with the NC group, the Rab7a knockout group significantly inhibited scratch healing (Figure 4(c)), indicating that Rab7a played an important role in mediating MSC migration.

### 3.6. Rab7a Knockout Weakens the Therapeutic Effect of MSCs on ARDS

**Figure 5:**
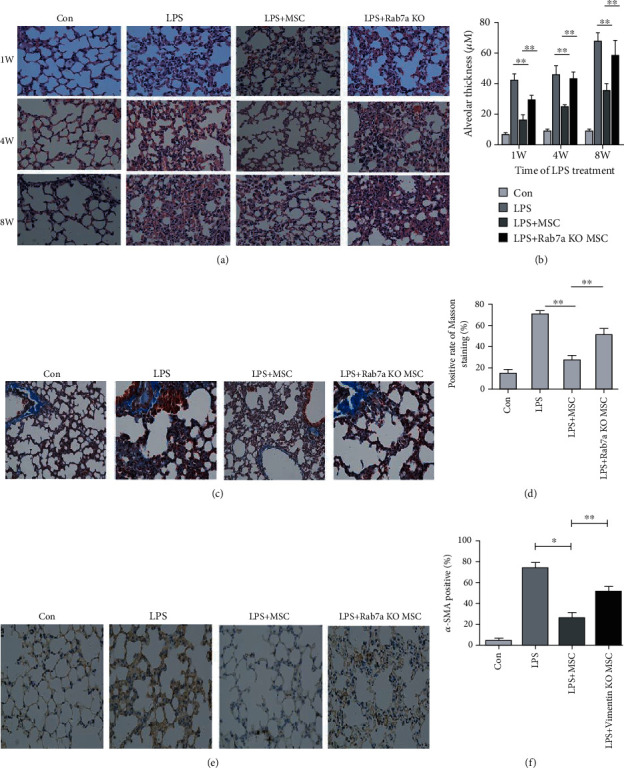
Rab7a knockout weakened the therapeutic effect of MSCs on ARDS. Two hours after the ARDS model was constructed, normal MSCs and Rab7a knockout MSCs were injected into the tail vein of mice. Left lung tissues of the mice were collected at 1 W, 4 W, and 8 W for H&E staining (a, b), Masson staining (c) at 8 W (d), and *α*-SMA immunohistochemical staining (e, f). Statistical results are represented as mean ± SD. ^∗^*P* < 0.05, ^∗∗^*P* < 0.01, *n* = 5.

We tested the effect of Rab7a knockout MSCs on ARDS. The Rab7a knockout MSCs were injected into mice with LPS-induced pneumonia, and the lung tissues of the mice were taken at 1 W, 4 W, and 8 W. H&E pathological examination showed that the knockout of Rab7a significantly weakened the therapeutic effect of MSCs in mice with LPS-induced pneumonia (Figures 5(a) and 5(b)). Masson staining (Figures 5(c) and 5(d)) and *α*-SMA staining (Figures 5(e) and 5(f)) results were consistent with H&E results, indicating that Rab7a knockout attenuates the therapeutic effect of MSCs on LPS-induced pulmonary fibrosis in mice.

## 4. Discussion

Alveolar epithelial injury is the main pathophysiological basis of ARDS. The alveolar type II epithelial cells remaining after injury can proliferate and differentiate into alveolar type I epithelial cells to repair lung injury, but, in moderate to severe ARDS, alveolar type II cells are severely damaged, and their number is insufficient for repair. For damaged alveolar epithelium, supplementation of exogenous repair seed cells is a viable option [13]. MSCs are stem cells with multidirectional differentiation potential derived from the mesoderm. Previous studies have shown that exogenous MSCs given in ARDS animal models can indeed repair damaged alveolar epithelium and even reduce the mortality rate, but the number of MSCs homing to the lungs is relatively small. Short lung retention time and low differentiation ratio remain difficult challenges faced to utilize MSCs in the treatment of ARDS [14]. Previous studies have shown that vimentin acts on cell migration, contraction, proliferation, protein synthesis, and protein synthesis.

Molecules that play an important role in gene expression, apoptosis, and mechanical force transmission [15] also play an important role in the migration of MSCs [16]. Therefore, the regulation of MSCs by targeting vimentin is expected to further improve the lung protection effect of MSCs. This study was verified by in vivo and in vitro experiments.

The current study found that mice with vimentin gene knockout had slower MSC growth than wild-type mice, and their differentiation status also differed. This suggested that there may be a considerable difference between the therapeutic effects of vimentin knockout mouse MSCs and wild-type mouse MSCs. The results confirmed that vimentin gene knockout weakened the therapeutic effect of MSCs in ARDS. Furthermore, we used GFP-labelled MSCs to observe their colonization in damaged lung tissues. The results showed that the homing effect of MSCs in vimentin knockout mice was severely weakened. We have been able to be relatively clear that the effect of vimentin on the therapeutic effect of MSCs is mainly reflected in the change of the migration effect of MSCs into damaged lung tissue. Presently, there have been many reports on vimentin-mediated MSC migration [17, 18], but the role of vimentin-mediated MSC regulation has not been clear. Although some studies, such as Dave et al. [19], reported that PIAS1 may play an important role in vimentin-mediated cell migration, MSCs are different from other cells. They are a kind of immature cell that is induced under different conditions. Under varying circumstances, their state will change, so the genetic law presented is often in an unstable state. Furthermore, MSCs secrete many cytokines, and many of these cytokines, such as IL-6, IL-8, and MCP-1, promote migration [20]. Some studies have reported that the effect of vimentin on cells not only promotes migration and proliferation but also has an important effect on the secretion of cytokines [21]. Therefore, vimentin-mediated MSC migration may be more complicated than the experimental results show and will require further investigation.

In addition to exploring the downstream mechanism of vimentin-mediated MSC migration, we investigated changes in the upstream signalling of vimentin. Many studies have shown that vimentin, as a skeletal protein, has many complex molecules that regulate its expression, which depends on different signal stimuli and different cell functions. In the regulation of cell migration, it is currently believed that Rab7a (a small molecule GTP binding protein) may be an important vimentin regulatory molecule. Some studies have shown that the role of Rab7a in regulating vimentin is not to affect its expression but to change the polarity of its molecules, thereby inducing cells to move in different directions. Studies have also shown that Rab7a can phosphorylate vimentin, thereby affecting the function of vimentin [9]. Obviously, the Vimentin-Rab7a pathway plays an important role in cell migration, but its mechanism is also very complicated. At present, there are no reports on the role of the Vimentin-Rab7a pathway on MSC migration. In our research, we observed the binding of Rab7a and vimentin in MSCs, for the first time, and used genetic blocking to prove that vimentin is indeed a downstream molecule of Rab7a. Interfering with the function of Rab7a also affected the migration of MSCs, affecting the therapeutic effect of MSCs in ARDS. This phenomenon also illustrates, however, that Rab7a may have a more important function than vimentin.

## 5. Conclusions

In summary, this study proved for the first time that Vimentin-Rab7a can enhance the migration ability of MSCs and affect its homing effect in damaged lung tissue, thereby mediating the treatment of ARDS. The findings of this study provide new insight into intervention targets for enhancing the therapeutic effect of MSCs in ARDS.

## Data Availability

The data used to support the findings of this study are available from the corresponding author upon reasonable request.
